# BrainSec: Automated Brain Tissue Segmentation Pipeline for Scalable Neuropathological Analysis

**DOI:** 10.1109/access.2022.3171927

**Published:** 2022-05-02

**Authors:** ZHENGFENG LAI, LUCA CERNY OLIVEIRA, RUNLIN GUO, WENDA XU, ZIN HU, KELSEY MIFFLIN, CHARLES DECARLI, SEN-CHING CHEUNG, CHEN-NEE CHUAH, BRITTANY N. DUGGER

**Affiliations:** 1 Department of Electrical and Computer Engineering, University of California Davis, Davis, CA 95616, USA; 2 Department of Pathology and Laboratory Medicine, University of California Davis, Davis, CA 95817, USA; 3 Department of Electrical and Computer Engineering, University of Kentucky, Lexington, KY 40506, USA

**Keywords:** Neuropathology, machine learning, medical image analysis, convolutional neural network, Alzheimer’s disease, dementia

## Abstract

As neurodegenerative disease pathological hallmarks have been reported in both grey matter (GM) and white matter (WM) with different density distributions, automating the segmentation process of GM/WM would be extremely advantageous for aiding in neuropathologic deep phenotyping. Standard segmentation methods typically involve manual annotations, where a trained researcher traces the delineation of GM/WM in ultra-high-resolution Whole Slide Images (WSIs). This method can be time-consuming and subjective, preventing a scalable analysis on pathology images. This paper proposes an automated segmentation pipeline (BrainSec) combining a Convolutional Neural Network (CNN) module for segmenting GM/WM regions and a post-processing module to remove artifacts/residues of tissues. The final output generates XML annotations that can be visualized via Aperio ImageScope. First, we investigate two baseline models for medical image segmentation: FCN, and U-Net. Then we propose a patch-based approach, BrainSec, to classify the GM/WM/background regions. We demonstrate BrainSec is robust and has reliable performance by testing it on over 180 WSIs that incorporate numerous unique cases as well as distinct neuroanatomic brain regions. We also apply gradient-weighted class activation mapping (Grad-CAM) to interpret the segmentation masks and provide relevant explanations and insights. In addition, we have integrated BrainSec with an existing Amyloid-*β* pathology classification model into a unified framework (without incurring significant computation complexity) to identify pathologies, visualize their distributions, and quantify each type of pathologies in segmented GM/WM regions, respectively.

## I. INTRODUCTION

The aging population is increasing all over the world: the population of people aged 60 or older is over 900 million and expected to be over 1,800 million by 2050 [1]. Alzheimer’s disease (AD), one of the neurodegenerative diseases that increase risks with age, resulted in more than 120,000 deaths in 2018, and the number of patients is predicted to rise to13.8 million in the U.S by the middle of this century [2]. There is an urgent need to better understand the underlying pathophysiology of this disease. To study and understand the progression of AD, experts visually inspect postmortem brain tissue slides and identify pathological hallmarks that are the gold standard for the diagnosis of AD [3]. Traditionally, experts compare these pathological hallmarks by carefully inspecting brain tissue on glass slides with the help of microscopes. Recently, Whole-slide Imaging (WSI) is an increasingly popular modality used to assess brain tissues with the help of a scanner, which converts the slides into a digital file (i.e. WSI) of ultra-high resolution [4]. WSIs can be easily viewed through computer softwares (such as Aperio ImageScope and/or QuPath [5]), these softwares also allow trained personnel to assess, identify, and quantify the morphologies with more computational approaches [6], [7].

There are many types of neuropathological hallmarks within the brain that aid in the diagnosis of neurodegenerative diseases, such as AD [8], and their neuroanatomic location and density can be critical to gain deeper insights into disease pathophysiology.^1^ One hallmark pathological feature of AD is the presence of extracellular Amyloid-*β* deposits in the form of amyloid plaques in human brain [3], [9], [10]. Often these deposits appear in grey matter (GM) while some have also been reported in white matter (WM) [11]. Quantitatively evaluating plaque distribution, especially withing specific anatomic regions, can be extremely time-consuming considering the wide existence of pathologies in brain tissue and the gigapixel resolution of WSIs, which prevents the analysis of a large amount of WSIs at one time [12]. Currently, neuropathologists approach plaque counting in a semi-quantitative approach, through the method proposed by the Consortium to Establish a Registry for Alzheimer’s Disease (CERAD) [13]. Adding segmentation of GM and WM on top of this process can turn the WSI analysis into an even more complex task. Therefore, to quickly gain more insights into the progress and manifestation of AD, it is necessary to construct an end-to-end framework that can automatically segment GM/WM regions, identify and quantify different types of pathologies, and visualize their distributions in GM and WM, respectively.

Recent convolutional neural networks (CNN) have been applied to WSIs primarily from other parts of human body (tongue, breast, lymph node, rectum [14], skin [15]), anatomical brain (instead of tissue level) [16], or images reconstructed from magnetic resonance imaging (MRI) and computerized tomography (CT) [17], [18]. Although these methods are helpful in the analysis of WSIs in various applications, there are several issues that limit the direct applications of these methods to WSIs for the analysis of AD pathologies.

**FIGURE 1.**
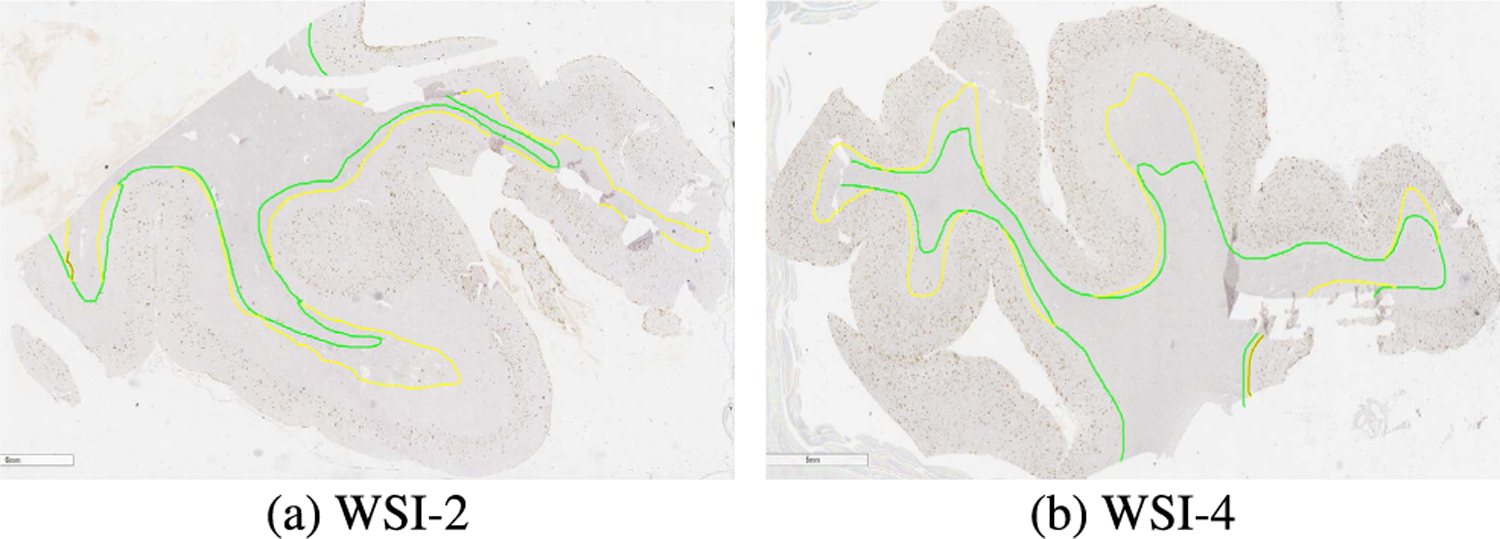
WSIs annotated by two trained personnel. (Green and yellow colors denote the two independent annotations.)

**FIGURE 2.**
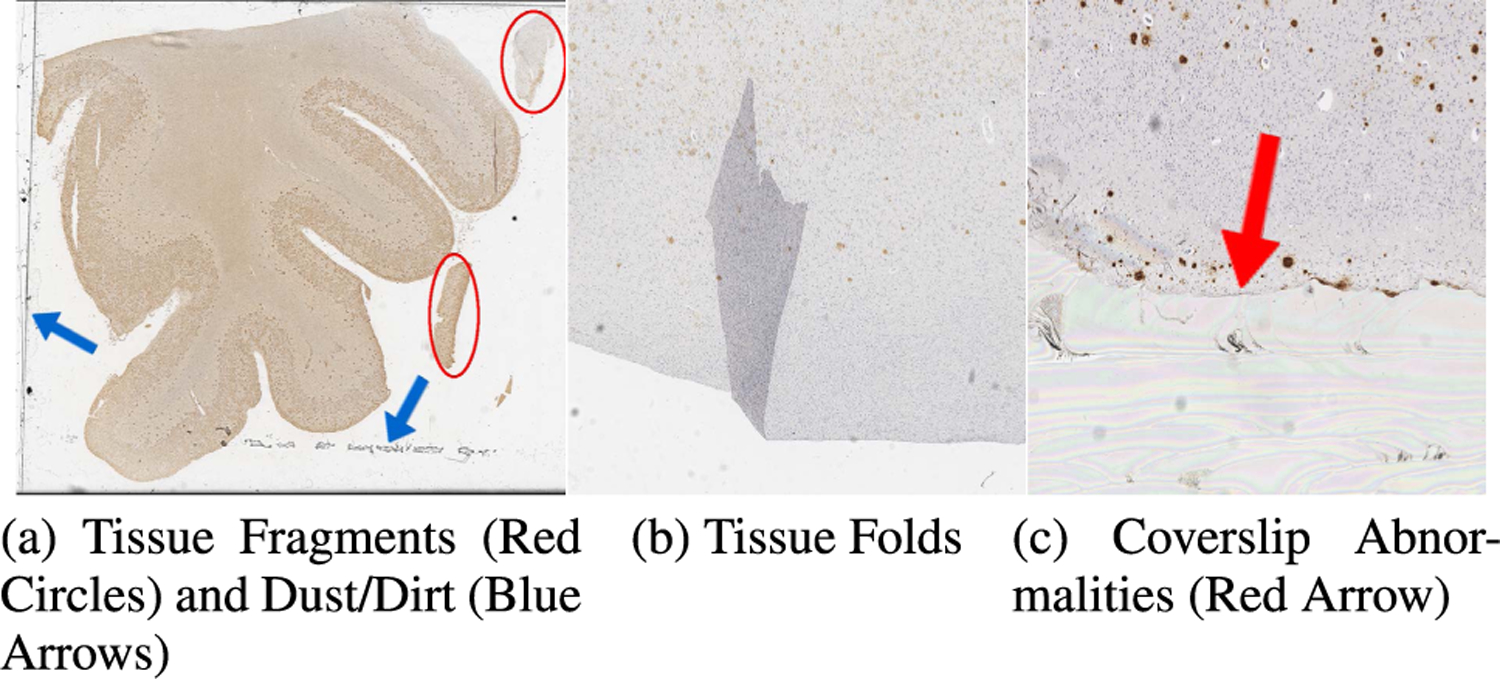
Sample artifacts in WSIs.

First, manual segmentation could be subjective and have inter-rater reliability issues. FIGURE 1 shows two examples, in each of which two trained personnel draw considerably different boundaries between GM and WM in the same brain WSIs. Second, our WSIs are scanned at ultra-high resolution to retain cellular level medical details (at least 20x magnification) and thus the average resolution of these WSIs exceeds 50, 000 by 50, 000 pixels, making the files very large and difficult to process in a timely manner. To solve this resolution issue, current methods can be divided into two categories: either downsample original images of ultra-high resolution or divide original images into small patches for separate processing [19]. Both the plaques and cells with sizes ranging from 20×20 to 100×100 in WSIs are distinguishable features of GM and WM, thus simply downsampling may make them less clear or even disappear [20], which results in limited predictive performance. On the other hand, we need to preserve the distributions and characteristics of plaques in WSIs for the study of AD. Therefore, in this paper we aim to focus on the patch-based approach. To provide a proof of concept for a workflow for GM/WM segmentation algorithm and to minimize the need for manual segmentation and tracings, we utilized 30 WSIs with annotations, far less than previous datasets used in [19]. We also utilized over 100 additional slides varying based on case as well as anatomic regions as additional hold out sets. Third, there are some unwanted artifacts in WSIs, such as tissue fragments, tissue folds, bubbles, and/or dust. A sample of these artifacts is shown in FIGURE 2. These artifacts set obstacles for segmentation methods to separate the background out accurately.

In this paper, we propose an entire pipeline used for segmenting GM/WM regions with a post-processing module that removes artifacts and unwanted fragments (as shown in FIGURE 2). Finally the pipeline generates XML annotations that can be displayed on the original WSIs. This pipeline is also able to incorporate exterior CNN modules that perform other tasks on the same region. We implement two baseline CNN models that are widely deployed in medical image segmentation problems, namely a fully convolutional network (FCN) [21] and a U-Net model [22], to perform pixel-wise segmentation of GM and WM. Then, we propose BrainSec, a novel mechanism that transforms the pixel-wise segmentation problem of gigapixel WSIs into a patch-wise classification problem in the first stage, then subsequently converts it back to the original segmentation task according to various resolution requirements of outputs. To further enhance the proposed workflow, we incorporated BrainSec into an existing pipeline that quantified amyloid pathologies in select brain areas [12]. To alleviate the prediction inconsistency issue in the first stage of BrainSec, we incorporate BrainSec with a Neural Conditional Random Field (NCRF) module to model the spatial relationships among neighboring patches, referred to as BrainSec+. BrainSec+ merges two modules into one by achieving similar effect of the post-processing module.

Our contributions can be summarized as follows:

- We establish an automated GM/WM segmentation pipeline, BrainSec. To omit the need for an additional post-processing step, we propose BrainSec+ based on BrainSec.
- We apply Gradient-weighted Class Activation Mapping (Grad-CAM) [23] to confirm what the model learns is explainable from a pathologic perspective by consulting a domain expert in neuropathology.
- We demonstrate BrainSec is consistently performing well over more than 180 WSIs (visually inspected by trained personnel) for a broader neuroanatomical evaluation.
- We show the compatibility of BrainSec into another neuropathology detection model by incorporating BrainSec into an existing pipeline to aid in quantification of neuroanatomic specific data on Amyloid-*β* plaques.
- After the integration, we propose an entire framework to quantify the number of neuropathological hallmarks and visualize their distribution in GM/WM regions.

## II. RELATED WORKS

### A. IMAGE PROCESSING-BASED SEGMENTATION

Although many methods [24], [25] have shown their success on Magnetic Resonance Images (MRIs), they may not be applicable to gigapixel-pixel WSIs considering the distinct imaging processes and visual differences between MRIs and WSIs [26]. For WSIs, Hiary *et al*. [27] built an automatic algorithm based on k-means clustering that worked on pixel intensity and texture features. Bug *et al*. [15] applied global thresholding at the mean value of the Gaussian blurred Laplacian of the greyscale image. In [28], stain concentration and porospity analysis was applied to the segmentation of H&E stained WSIs. Although these methods are computational efficient, the segmentation results are very limited due to inter and intra-variations in staining and color contrast, resulting in general failure for the above mentioned methods on hold-out test sets [29]. Additionally these methods have only been tested on non-brain WSIs like breast, tongue and skin, so their applicability and feasibility on our brain WSIs has yet to be determined. In this work, we first investigated the above methods on our slides but they may not generalize well on hold-out test sets and need to be fine-tuned for heterogeneous settings (e.g., different scanners, different brain regions, etc.). Hence we turn our attention to deep learning-based methods to seek for a more generalizable approach.

### B. DEEP LEARNING-BASED SEGMENTATION

In recent years, deep learning methods have gained vast popularity in image segmentation problems in pathology involving use of WSIs. In [30], CNNs and Image Processing methods are compared for segmenting invasive ductal carcinoma in breast tissue slides. Breast tissue WSIs were also used by [31] to achieve segmentation of tumor tissue through use of DCNNs. In [32], a ResNet model pretrained on ImageNet was tuned through a Transfer Learning approach to segment the mitotic cells in pre-processed breast tissue WSIs. Recently, in [33] a pipeline powered by VGG16 was used to segment cancer tissue in bladder WSIs and also grade the severity of cancerous tissue.

Besides these CNN methods, FCN [14] and U-Net [29] based architectures are predominant choices in medical image segmentation problems [22], [34]. The study from [35] is an example of U-Net being applied for nuclei segmentation from glioma patients’ WSIs. In [36] we see a modified version of U-Net, Y-Net, achieve state-of-the-art segmentation of whole slide breast cancer biopsy images. An multi-scale U-Net was also used by [37] to segment whole slide prostate images in order to identify and grade cancerous tissue. Some studies have used U-Net in medical image segmentation involving the brain, but using data different than WSI [38]–[40]. So far, only one study to our knowledge has investigated these architectures on GM/WM segmentation in gigapixel pathology images [41]. However, the study used a total of 49 Tau stained WSIs and only FCN architecture was used. Here, we examined multiple architectures and compare their performance. The details of how we implement them as benchmarks are as following.

#### 1) FCN

When applied to tissue segmentation of histopathological WSIs, FCN outperforms traditional methods and achieve less outliers and more stable results [14]. The most recent FCN model we explored is based on the architecture and hyper-parameters in [14]. We used the FCN-8s model from the original FCN paper [42], which combined predictions from the final layer, pool4 layer and pool3 layer at the stride of 8. The FCN architecture contains 7 convolutional layers. The first two layers have the filter size 5 × 5. The third and fourth layers have filter size 3 × 3. The fifth layer has the size of 11 × 11. The last two layers have 1 × 1 convolution, which are equivalent to the fully connected layers. The number of filters at each layer are 16, 32, 64, 64, 1024, 512 and 2 respectively. Filter size of 2 × 2 and stride of 2 are used to all the max pooling layers. The number of max pooling layers is based on original FCN paper [42], which are inserted after first five convolutional layers to reduce memory requirements of the network. The batch normalization layer and sigmoid activation layer are added after every convolutional layer for the regularization and convergence purposes. Drop out layer with drop out rate of 0.2 is added after every convolutional layer to reduce the probability of overfitting. The batch size is 128.

By benchmarking the results of different loss functions, we chose the combination of the categorical cross entropy loss and focal loss for FCN [43], [44]. By setting the weight of different output classes to be 1 and training FCN on categorical cross entropy loss and then on focal loss, the average validation results could outperform the model that used either categorical cross entropy or focal loss alone (weighted or unweighted).

#### 2) U-NET

Although there are many versions of U-Net architectures, most are designed for natural image segmentation instead of gigapixel WSIs. In this study, we select the most updated U-Net model modified for WSIs [29] as our baseline. The contracting path of the network has four convolution levels. Each level consists of two consecutive 5 × 5 convolution operations (zero-padded convolutions) with Exponential Linear Unit (ELU) [45] activation functions, followed by a 2 × 2 max-pooling operation with stride 2 for down-sampling. At each down-sampling level, the number of feature channels is doubled (32 − 64 − 128 − 256 − 512). The expansive part of the network also has four convolution levels. Each level consists of a 2 × 2 transposed convolution that halves the number of feature channels, a concatenation with the corresponding contracting level output, and two 5 × 5 convolutions with ELU activation. To speed up learning and provide some regularization effect, we used batch normalization [46] after each convolutional layer. We also incorporated drop-out [47] after the first convolutional layer at each level, both in the contracting and the expansive path. A final 1 × 1 convolution with 3 output channels is then used to map the last feature map to the class prediction output.

Prior to the training, all convolution kernels were initialized using *He’s uniform* initialization [48]. We used the *Adam* optimizer [49] with an initial learning rate of 0.001. The *α* parameter for the ELU activations was set to 1.0, the drop-out rate was 0.2, and the batch size was 16. To diminish the effect of class-imbalance issue in our dataset, we weighted the categorical cross entropy loss function by the inverse of class frequencies in the training dataset.

A major drawback of U-Net based methods is they require computationally intensive operations on GPUs and take a great deal of processing power with very trivial gain in performance [50].

## III. DATASETS

### A. 30-WSI DATASET

To provide quantitative results and initially test, train, and validate our pipeline, we first evaluated a 30 Whole Slide Images (WSIs) dataset annotated using QuPath [5]. These WSIs were from formalin fixed paraffin embedded 5um section of the temporal cortex stained with an antibody directed against Amyloid-B (4G8, BioLegend (formally Covance, San Diego, CA). These WSIs were digitized by Aperio AT2 at up to 40× magnification, the resolution is nearly 60, 000 × 50, 000 pixels each on average. These 30 WSIs can be split into two sets: one set includes 18 cases that had a pathologic diagnosis of Alzheimer’s disease while the other set (12 cases) lacked a pathologic diagnosis of Alzheimer’s disease but may have other diseases, such as vascular disease (Non-Alzheimer’s disease, NAD). The ethnoracial make up of the cohort was 3 Hispanics (10%), 5 African Americans (17%), and 22 non-Hispanic White (73%) descendants.

These studies utilized tissues only from human postmortem. Only living subjects are confirmed as Human Subjects under federal law (45 CFR 46, Protection of Human Subjects). All participants or legal representative approved informed consent during the life of the participant as part of the University of California Davis Alzheimer’s disease Center program. The data collection process followed current laws, regulations and IRB guidelines. All of these 30 WSIs had been de-identified, lacking personal health information like names, social security numbers, addresses, and phone numbers. To further protect data confidentiality, we named the AD cases as WSI-1 to WSI-18 and NAD cases as WSI-19 to WSI-30.

### B. TRAINING DATA PREPARATION

To deal with the issue of ultra-high resolution, we use Pyvips Library [51] to set up image processing pipelines on original-resolution WSIs instead of directly manipulating these slides. Hence, we avoided loading the entire image into memory at once for processing because Pyvips can stream the image in parallel from the first step to the last step of pipelines simultaneously. The proposed approach in [20] manually selected regions from GM, WM, and background and cropped small tiles from these regions separately. This manual action could result in limited variety of datasets and involve human interventions. As we have pixel-wise annotations on GM and WM, we randomly cropped patches 256 × 256 from the slides in the training set (20 slides) and labeled them as follows: each patch was labeled with the category of the central pixel of that patch tracing back to our pixel-wise ground truth. After this, we obtained around 310k patches from GM, 100k patches from WM, and 300k patches from background. In our experiments, 20 WSIs (12 AD cases and 8 NAD cases) were randomly selected for training and validation while the remaining 10 WSIs (6 AD cases and 4 NAD cases) were used for **hold-out** testing and inference. The data split was fully random as GM region and WM region are in the same level of magnitude (the ratio between GM/WM region in one slide ranges from 3:1 to 1:1).

For the annotation process, we first invited one trained personnel (K.M) for drawing the boundary between GM and WM. Then we sent these annotations to an expert (B.D) for checking and making the final annotation. We use the annotation verified by the expert as ground truth.

### C. 130-WSI AND 52-HETEROAREA DATASETS

In order to rigorously test the performance of our segmentation model, two additional datasets were prepared from available convenience samples. The 130-WSI dataset contained temporal WSIs from 130 new cases not included in the 30-WSI dataset. This dataset aimed at testing our model on as many new WSIs as possible to check if performance is affected by applying it to an unseen, larger cohort. There were 13 NAD cases and 117 AD cases in this set. The ethnoracial make up of the cohort was 13 Hispanics (10%), 15 African Americans (11.5%), 6 Asian Americans (4.6%), 1 Other (0.8%) and 95 non-Hispanic White (73.1%) descendents.

The other additional dataset, 52-Heteroarea, contained 52 new WSIs from two brain areas not seen by the model: areas from frontal and parietal lobes. The goal of this dataset was to test our model performance on brain areas different than the temporal region. The 52-Heteroarea contained 26 cases, with 13 cases featured in the 130-WSI dataset, 2 cases featured in the 30-WSI dataset and 11 new cases. From each of the 26 cases, 2 WSIs were acquired, one from each unseen brain areas (frontal and parietal cortex sections). The summary of the information on each of the three datasets used can be seen on TABLE 1. For both the 130-WSI and the 52-Heteroarea datasets the procedure for staining, scanning and preprocessing was the same as the 30-WSI dataset. All protocol regarding de-identification and confidentiality followed by the 30-WSI dataset was also followed for the additional two datasets. These additional datasets were treated as hold-out sets, no ground truth GM/WM annotations were collected. The goal was to perform a proof of concept by visually inspecting the BrainSec prediction results (background vs. GM vs. WM).

## IV. METHODOLOGY

In this section, we first introduce BrainSec, a GM/WM segmentation pipeline. Then we present its integration into a patch-based pathology classification model [12] and propose a framework to automate the quantification of pathologies in GM/WM regions in a scalable way and visualize the distributions of each type of pathology. We release our codes on https://github.com/ucdrubinet/BrainSec.

### A. BRAINSEC

BrainSec has two modules: CNN module and post-processing module. CNN module consists of two stages: transform the problem to patch-based classification and convert back to pixel-wise segmentation of WSIs.

#### 1) PATCH-BASED CLASSIFICATION

In this stage, we transform the pixel-level segmentation problem into a patch-based classification problem. For image classification tasks, a myriad of different CNN architectures have been proposed, such as AlexNet, VGG, ResNet [52] in the past few years. While design motivations of each architecture are greatly different, there is increasing evidence showing that the features extracted from these state-of-the-art architectures are quite akin and their improvements gradually become trivial and start to converge [53]. To produce reasonable results with relatively minimized complexity, we decide to select a simple architecture to pursue the trade-off between complexity and performance. In [54], He *et al*. showed their ResNet architecture has fewer filters and lower complexity but achieves similar performance compared to other state-of-the-art architectures. As such, we selected the ResNet-18 architecture described in [54] as our backbone network and the fundamental basis of our methods. We selected 1 × 1 convolution skip connection instead of identity skip connection in our backbone.

We modified ResNet-18 by redefining the last fully connected layer to output three categories: GM, WM, and the background of tissue slides. As we transform the segmentation problem into a classification problem in this stage, the function of ResNet-18 here is to extract the features from each patch and classify its corresponding category. We adapted pre-trained parameters except for the last layer from ResNet-18 trained on ImageNet because it has already shown the ability of extracting useful features from natural images. We introduced categorical weights to the multi-class cross-entropy loss based on the inverse of class frequencies in our training set. Specifically, as we have around 310k patches from GM, 100k patches from WM, and 300k patches from background in our training set, the weight ratio of GM:WM:background is 1:3:1 (inverse of class frequencies). We used *Adam* optimizer [49] and the initial learning rate is 0.001. We adopted the automatic early stopping criteria by saving checkpoints for each epoch for both training and validation sets, subsequently selecting the well-trained model automatically. The batch size was set as 16.

#### 2) PIXEL-WISE SEGMENTATION

**FIGURE 3.**
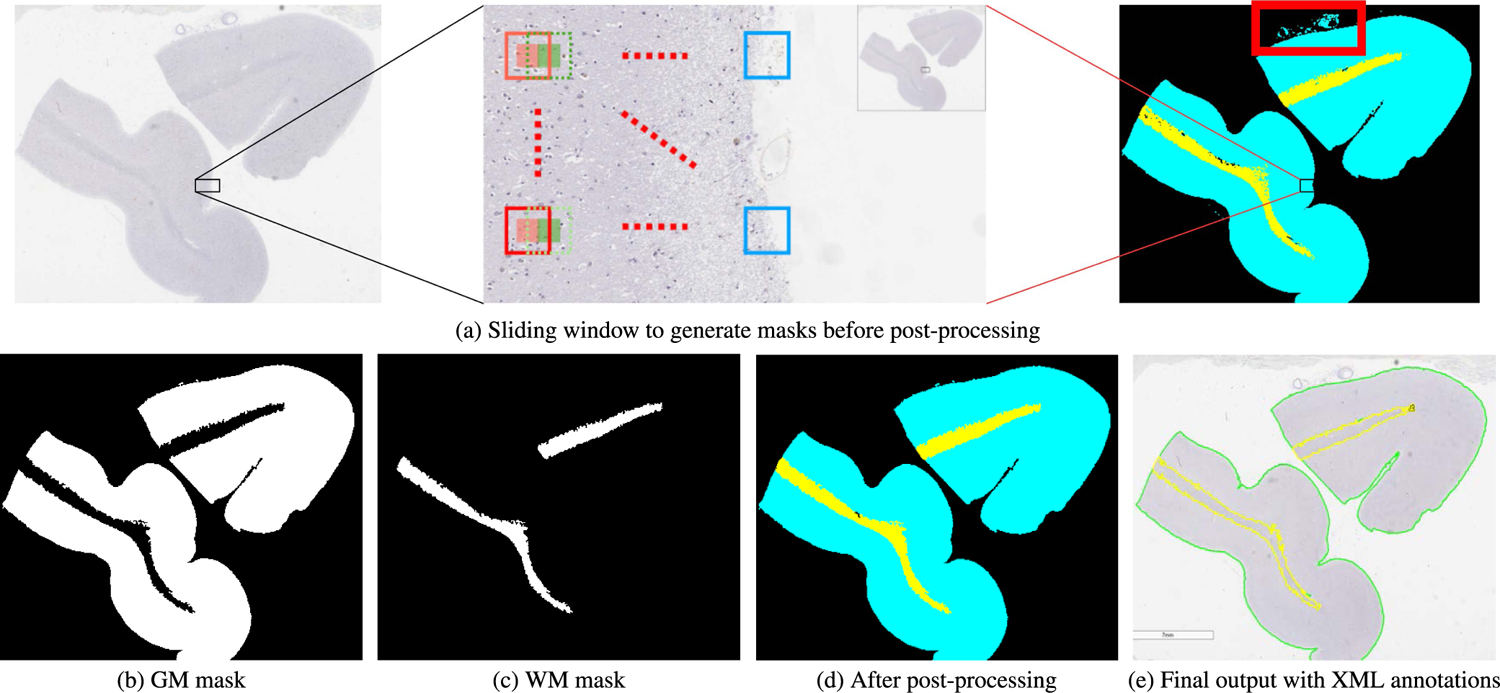
Segmentation pipeline and output of XML annotations. The red box indicates the tissue fragments that needs to be removed by the post-processing module.

In this stage, we utilize the results from the classification task to construct pixel-wise segmentation output. In the previous stage, each patch is classified to a corresponding output class (GM, WM, or background) using our modified ResNet-18 model. In order to achieve pixel-wise segmentation results, we use a sliding window approach to extract each patch until the whole image is fully covered. The resolution of output masks will be decided by the step size. For example, as shown FIGURE 3a, step size here is set as 128 while the patch size is 256. The red patch in FIGURE 3a is fed into our modified ResNet-18 first, subsequently receives the prediction category. After that, if output masks are required to be the same size of original WSIs, all pixels in the central area (red block) with the size of step_size × step_size (128 × 128 here) will be classified as the same category predicted by the modified ResNet-18 model. Then we make a step forward with the step size to the green patch with dotted line and repeat the same action, so the green block next to the red block will be labeled with the same category. By repeating this action, after the sliding window traverses the whole image, we can get segmentation masks for GM, WM (as shown in FIGURE 3b, c) and background separately. As such, we can see the accuracy of output masks is determined by step size: if step size is larger, outputs will be less accurate around boundaries but inference complexity will be reduced; if step size is smaller, outputs will be more accurate but inference complexity is also increasing. Hence, BrainSec provides flexibility to achieve different trade-offs between performance and complexity. On the other hand, if the goal is to construct a framework for pathology quantification and visualization (as shown in Section IV–C), the step size should be correlated with the size of a pathology. In the example of Section IV–C, we expect the size of a sliding window could be adequately larger than the size of an Amyloid-*β* deposit thus selecting it as 128 to identify and precisely locate each pathology. The step size can be altered for other types of pathologies.

#### 3) POST-PROCESSING

We design a post-processing module to refine our segmentation output masks and remove tissue residues in our WSIs. The full-resolution predicted outputs are down-sampled by a factor of 16 (1/4 in width and 1/4 in height) to reduce computational complexity. Two consecutive area openings are applied to remove fuzzy predictions of GM and WM with pixel area < 20, 000 and an area closing is applied to remove fuzzy prediction of background with pixel area < 12, 500. Then, small tissue residues with area smaller than 5% of the WSI area are removed. In the end, a morphological opening with a disk-shaped kernel with radius of eight is applied to smooth the boundary and the output was up-sampled back to the original resolution. The kernel sizes used for morphological opening and the size threshold for pixel areas removed are obtained empirically. FIGURE 3a, 3d is an example showing the difference between before and after the post-processing method is applied to remove tissue fragments.

In addition to these steps, we also generate XML annotation files of GM and WM segmentation boundaries by finding contours of GM and WM segmentation masks respectively as shown in FIGURE 3e. These XML files are in the same format of our ground truth annotations and can be visualized and displayed on original-size WSIs using Aperio ImageScope. They are also downsampled by 50× to smooth out the boundary for faster visualization.

### B. BRAINSEC+

**FIGURE 4.**
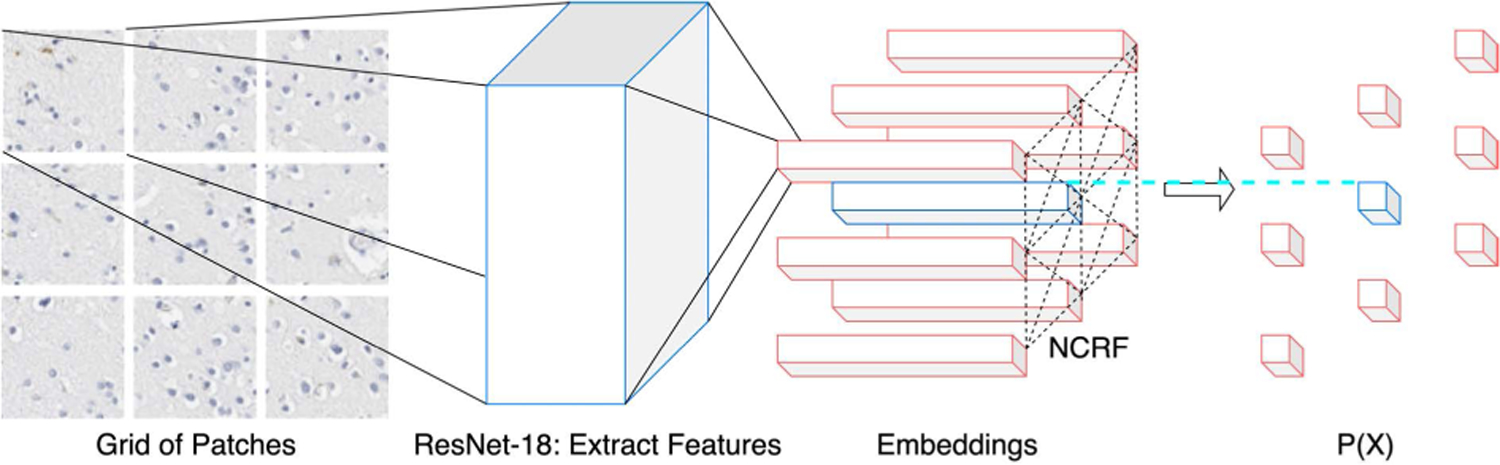
NCRF architecture.

The post-processing module in Section IV–A3 is effective for refining the segmentation output of BrainSec. However, it still requires parameter tuning, which may prevent it from the wide-adoption of various pathology datasets. In this section, we propose BrainSec+, which is built upon BrainSec by using similar mechanism and backbone as BrainSec while integrating a Neural Conditional Random Field (NCRF) layer that incorporates spatial relationships among neighboring patches to alleviate the need for a post-processing module. Conditional Random Field (CRF) BrainSec+ (as shown in FIGURE 4) consists of two components: ResNet-18 component is used to extract features and encode each patch as an embedding (a vector representation with a fixed length) by taking a grid of patches as input; NCRF layer is used to model spatial correlations among the grid of patches and provide an output of the probability of the central patch in the grid.

#### 1) SPATIAL CORRELATIONS WITH NCRF

NCRF is a probabilistic graphical component that can take the context of the neighboring of a certain target into account to model the spatial information. Given a grid of patches, we define a random field *X* = {*X*_1_, *X*_2_, …, *X*_*N*_} as the random variables related to each patch, where *N* is the number of patches in the grid, e.g. 9 for a grid of 3 × 3. Each random variable represents a label from the set of {*GM*, *WM*, *background*}, conditioned on observations *I* = {*I*_1_, *I*_2_, …, *I*_*N*_}, where *I* is the embedding of each original patch extracted by the CNN component. This set of label is defined as *x* = {*x*_1_, *x*_2,_ …, *x*_*N*_}. Therefore, different from the CRF methods for pixel labeling, *I* is not a set of pixel attributes such as RGB color values or intensity but a set of patch descriptors: CNN features from each patch. The distribution of (*X*, *I*) can be a CRF if the random variables of set *X* conditioned on observations *I* meet requirements of Markov property. Hence structured predictions can be transformed as:

(1) $$y*=\underset{x\in X}{\text{arg max }}P(X|I)$$

where *P*(*X*|*I*) is defined as a Gibbs distribution:

(2) $$P(X|I)=\frac{1}{Z(I)}\text{exp}(-E(X|I))$$

In a fully-connected CRF [55], the energy of Gibbs can be written as:

(3) $$E(X|I)=\underset{i}{\sum }\psi_{u}\left(x_{i}|I_{i}\right)+\underset{i<j}{\sum }\psi_{p}\left(x_{i},x_{j}|I_{i},I_{j}\right)$$

where *i*, *j* ∈ {1, 2, …, *N*}. The energy function *E*(*X*|*I*) is the summation of two terms: a unary potential *ψ*_*u*_(*x*_*i*_ |*I*_*i*_) and the pairwise potential *ψ*_*p*_(*x*_*i*_, *x*_*j*_|*I*_*i*_, *I*_*j*_). The unary potential can be considered an initial estimate of the cost of patch *i* with its label *x*_*i*_ given its corresponding embedding *I*_*i*_. Similarly, the pairwise potential estimates the joint cost of patch *i*, *j* with their label *x*_*i*_, *x*_*j*_ given their corresponding embeddings *I*_*i*_, *I*_*j*_. In other words, the pairwise potential is a measure of relationships between two patches and is defined over every pair of patches in the grid. In [56], they chose cosine similarity to define the pairwise potential because cosine similarity could measure the similarity of each embedding. Hence it can encourage the CNN to produce similar embeddings when the images look similar. Cosine similarity here is not used directly for the segmentation problem, but to encourage the model to have consistent predictions on similar patches. We select cosine similarity among the choice of other metrics due to its effectiveness and simplicity (i.e., can be incorporated without introducing too much computation complexity). The definition of cosine similarity in this case is:

(4) $$\psi_{p}\left(x_{i},x_{j}|I_{i},I_{j}\right)=H\left(x_{i}=x_{j}\right)\cdot w_{i,j}\left(1-\frac{I_{i}\cdot I_{j}}{\left\|I_{i}\right\|\cdot \left\|I_{j}\right\|}\right)$$

where ***H***(*I*_*i*_ = *I*_*j*_) is an indicator function that checks the label compatibility between *X*_*i*_, *X*_*j*_. By using the cosine similarity, the spatial correlations between a pair of patches can be modelled by encouraging lower cost in the case that *X*_*i*_, *X*_*j*_ are assigned with the same label if the embeddings *I*_*i*_, *I*_*j*_ are similar. *w*_*i*,*j*_ is a single, trainable parameter which the authors claimed related to spatial distance between patches [56].


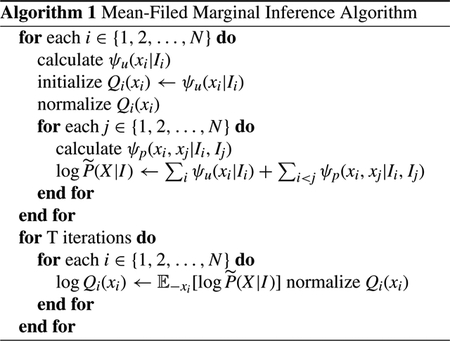


#### 2) END-TO-END BACK-PROPAGATION

To compute the cross-entropy loss with ground truth labels, we need to get the marginal distribution of each patch label *x*_*i*_ so the standard back-propagation algorithms could be applied to achieve end-to-end training. A mean-field approximation method was proposed in [57] to approximate maximum posterior marginal inference. As exact marginal inference is intractable, by using the mean-field approximation, the original CRF distribution *P*(*X*) can be transformed to *Q*(*X*) which is obtained by the product of independent marginal distributions, as shown in (5):

(5) $$P(X)\approx Q(X)=\overset{N}{\underset{i}{\prod }}Q_{i}\left(x_{i}\right)$$

Here *Q*(*X*) is a simpler distribution compared to *P*(*X*). The KL divergence between *Q*(*X*) and *P*(*X*) is shown in (6).

(6) $$KL(Q(X)∥P(X))=\underset{i}{\sum }Q\left(x_{i}\right)\text{log}\frac{Q\left(x_{i}\right)}{P\left(x_{i}\right)}$$

The first item is the negative of entropy while the second item being cross-entropy. Assuminge $\tilde{P}(X|I)=\text{exp}(-E(X|I))$ as the unnormalized CRF distribution, we can derive the cross-entropy item as shown in (7).

(7) $$\underset{i}{\sum }Q\left(x_{i}\right)\text{ log}P\left(x_{i}\right)=\underset{i}{\sum }Q\left(x_{i}\right)\text{log}\frac{\tilde{P}\left(x_{i}\right)}{Z}\;\;=\underset{i}{\sum }Q\left(x_{i}\right)\text{log }\tilde{P}\left(x_{i}\right)-\sum Q\left(x_{i}\right)\text{log }Z\;\;=\underset{i}{\sum }Q\left(x_{i}\right)\text{log }\tilde{P}\left(x_{i}\right)-\text{log }Z$$

For minimizing KL divergence, − log *Z* can be dropped as this is an additive constant. This leaves us with the following optimization problem:

(8) $$\text{arg min}K_{L}(Q(X)∥P(X))\;\;=\text{arg min}\sum Q_{i}\left(x_{i}\right)\left(\text{log }Q_{i}\left(x_{i}\right)-\text{log }\tilde{P}\left(x_{i}\right)\right)$$

As KL divergence cannot be negative, the minimum of Equation 8 can be derived as:

(9) $$\text{log }Q_{i}\left(x_{i}\right)=E_{-x_{i}}[\text{log }\tilde{P}(X)]+\text{ const }$$

This is the update equation for each marginal distribution *Q*_*i*_(*x*_*i*_), where $E_{-x_{i}}[\text{log }\tilde{P}(X)]$ refers to the expectation of log $\tilde{P}(X)$ on all *x* except *x*_*i*_.

In summary, our mean-field marginal inference algorithm is shown in Algorithm 1.

### C. PATHOLOGY QUANTIFICATION & VISUALIZATION WITH BRAINSEC

**FIGURE 5.**
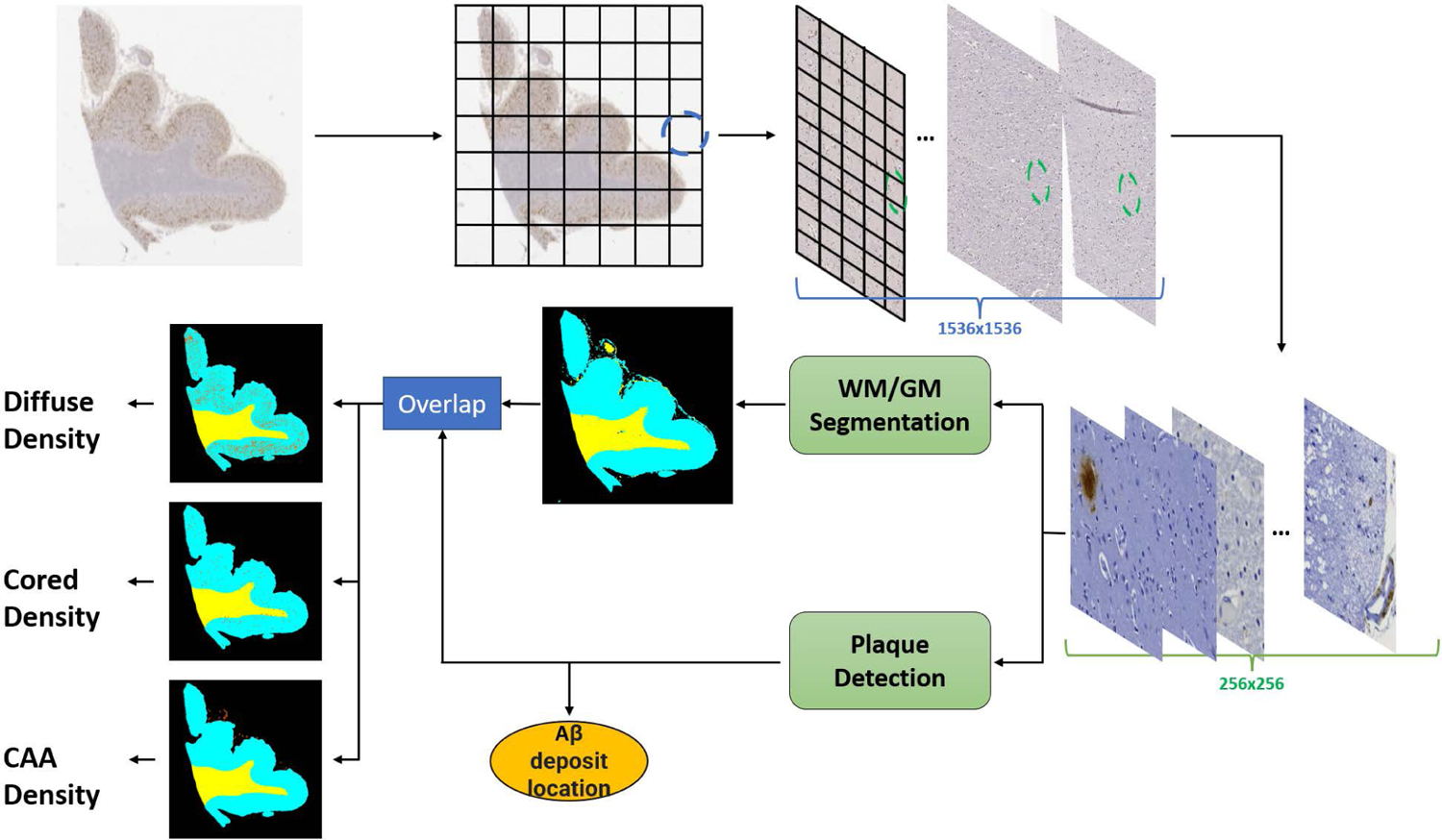
Overview of a pathology quantification model and visualization framework from Tang *et al*. [12] (indicated by yellow oval) integrated with BrainSec. A*β* in the figure refers to Amyloid-*β*.

As BrainSec is more computationally efficient, we use this as one example on to illustrate its compatibility with other patch-based pathology models. Based on BrainSec and the pathology model [12], we propose an entire framework (as shown in FIGURE 5) to quantify each type of Amyloid-*β* deposit and visualize distributions in GM/WM regions. We denote BrainSec in this case as *g*(·). *f* (·) [12] was trained over 33,111 tiles at 256 × 256 pixel level to distinguish Amyloid-*β* in the form of diffuse plaques, cored plaques, and cerebral amyloid angiopathy (CAA).

#### 1) SLIDING WINDOW FOR INFERENCE

A sliding window method is applied to visualize the distribution and location of Amyloid-*β* pathologies from a global view by generating WSI heatmaps of predictions. These heatmaps plot the location of each plaque predicted by *f* (·) up to the full WSI view. A stride size of 16 pixels is used to walk through the WSI using the sliding window, resulting in the confidence heatmaps at a fraction of the resolution of original WSIs. This is helpful for reproducibility without excessive loss of information when implemented on modern devices equipped with Graphics Processing Units (GPUs). For each WSI, it will generate three confidence heatmaps corresponding to cored, diffuse, and CAA, separately. After that, the cleaning and blob labeling are applied to the heatmaps, subsequently, specific thresholds are applied to each plaque type, which converts the heatmaps to binary masks: probabilities below the threshold would be converted to zero, and above would be one. To study the distribution of different plaque types in GM and WM, *g*(·) is incorporated into this framework to generate prediction heatmaps of GM and WM by using the same sliding window of stride size at 16 pixels to guarantee the output of *f* (·) is of the same size of the output of *g*(·) so that they can be pixel-wise overlapping. The heatmap of GM/WM will be displayed with the heatmap of plaques as shown in FIGURE 5: we visualize the distribution of each type of amyloid pathology, separately.

#### 2) PLAQUE QUANTIFICATION

The plaque quantification algorithm is summarized in Algorithm 2. A zero vector *P* with the shape of 1 × 6 is set up to record the number of each type of plaques (CAA, cored, diffuse) in GM and WM regions, separately, as the initialization. Then each WSI is normalized and tiled into 1536 × 1536 pixel images. After that, the sliding window is applied to extract a patch *P* at 256 × 256 pixels in an order from left to right, top to bottom. The patch will be the input of *f* (·) and *g*(·) to output the predictions in terms of both the type of deposit and the category of the region. These predictions are converted into a temporary one-hot vector *I*, which is added to *C* until the sliding window walks over the entire WSI. The final output is the vector *C* containing all quantification information of each type of plaques in GM and WM. All hyper-parameters involved in this component are strictly following the settings of pathology detection model [12] as our goal is to show the distribution of pathologies in GM and WM.


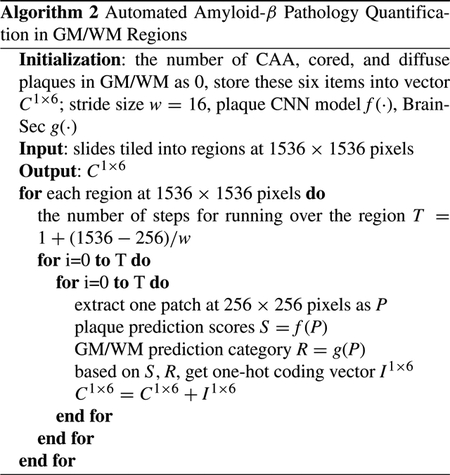


## V. EXPERIMENTS

### A. QUANTITATIVE RESULTS

Since our goal is pixel-level GM/WM segmentation, we used three standard segmentation metrics — IoU score [58], DICE coefficient [59] and F1-score [60] to comprehensively compare segmentation masks from different methods.

#### 1) IOU SCORE

IoU score [58], as shown in (10) is to measure the overlapping amount between the ground truth mask and the predicted mask.


(10) $$\text{IoU}=\frac{\text{ Area of Overlap }}{\text{ Area of Union }}.$$

The slides shown in FIGURE 3 and FIGURE 7 clearly indicate the inherent heterogeneity of brain tissues among different WSIs, where GM/WM regions have a great variety of sizes and shapes. Considering its variety of shapes, we also select standard deviation (STD) to quantify how consistent and robust our methods are on different hold-out WSIs. Larger standard deviations indicates more performance inconsistency among heterogeneous slides. FCN achieves inferior performance compared to U-Net (its mean IoU is more than 20% lower than BrainSec and U-Net), which is similar to the results reported in [61], [62]. Hence we will only compare BrainSec/BrainSec+ with U-Net in the subsequent sections.

From TABLE 2, BrainSec/BrainSec+ achieved the highest IoU and lowest standard deviation in the majority of indexes, which indicated that BrainSec/BrainSec+ was more stable and robust to a new hold-out test set compared to FCN and U-Net. Specifically, the mean IoU of BrainSec achieved more consistent segmentation results (less uncertainty) for both AD and NAD cases, since the difference between that of AD and that of NAD is only around 0.1% while there was more disparity in the confidence interval for AD vs. NAD cases for U-Net. The increased uncertainty in the segmentation results for AD compared to NAD is expected due to the existence of large amounts of plaques in AD cases.

TABLE 2 shows that BrainSec achieved 1.6% higher than U-Net in the mean IoU of AD cases, with smaller confidence intervals. Both BrainSec and U-Net achieve compatible IoU for NAD cases, but there was less uncertainty (smaller confidence interval) for BrainSec. Therefore, we can conclude that BrainSec shows stronger distinguishing ability in GM and WM among AD cases where boundaries may be more difficult to distinguish due to disease processes. BrainSec+ further improves BrainSec’s ability in WM among NAD cases and removes the need for post-processing.

#### 2) DICE COEFFICIENT

Besides the IoU score [58], we selected DICE coefficient [59], another popular metric used in segmentation problems, to further evaluate the performance of BrainSec and BrainSec+. DICE coefficient [59], as shown in (11), is calculated by doubling the overlapping areas and dividing it by the total number of pixels in both areas.


(11) $$\text{DICE }=\frac{2\times \text{ Area of Overlap }}{\text{ Total Sum of Pixels in All Areas }}$$

The results are summarized in TABLE 3. BrainSec/BrainSec+ was consistently superior to both FCN [14] and U-Net [29].

#### 3) F1-SCORE

In addition to the IoU score [58] and DICE coefficient [59], we also select F1-score as a third metric to compare the quantitative results. The results are summarized in TABLE 4. BrainSec/BrainSec+ can perform better performance especially in WM compared to FCN [14] and U-Net [29].

### B. TRAINING CONVERGENCE OF BRAINSEC

**FIGURE 6.**
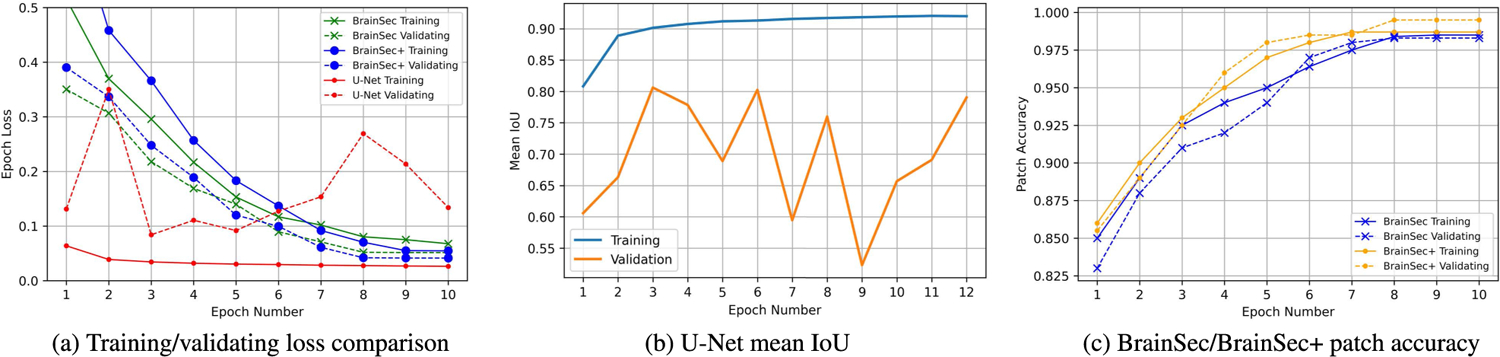
Training process comparison: visualization of the trends of training and validation comparing BrainSec/BrainSec+ with U-Net.

To compare the robustness of BrainSec/BrainSec+ with U-Net, we also analyzed the characteristics of training process of BrainSec/BrainSec+ and U-Net as shown in FIGURE 6. During our experiments, we found the epoch loss of U-Net on the validation set tended to oscillate with the epoch numbers while the loss on the training set strictly decreases (FIGURE 6a). However, the validation loss of BrainSec/BrainSec+ has the similar tendency compared to its training loss (both of them are strictly decreasing).

To further analyze the oscillation issue of U-Net and compare it with BrainSec/BrainSec+, we selected mean IoU of training and validating as performance metric as U-Net is a pixel-level architecture that outputs pixel-wise masks for GM and WM. FIGURE 6b shows its mean IoU results on the validation set tend to oscillate across the number of trained epochs while the results on the training set strictly increase (FIGURE 6b). Besides, the mean IoU of training was over0.9 while it could be 0.55 in the validation set at 9th epoch of training. Therefore, we performed five-fold cross-validation on the training and validation set to determine an optimal early stopping point for the number of training epochs. Then we trained the model for that number of epochs on the combined training-validation set and evaluated on the test set. The number of training epochs we obtained is 5 for U-Net.

On the other hand, BrainSec/BrainSec+ is a patch-level classification problem, so we chose patch-level accuracy to examine the training/validating process. FIGURE 6c shows the classification accuracy of BrainSec/BrainSec+ continues to increase for both training and validation sets. Both achieve 0.96 accuracy or above after 6 epochs, indicating that their results are very close without any oscillations or instability.

### C. COMPUTATION COMPLEXITY

For our pathology quantification framework, we have two components. The pathology identification component [12] includes three major steps: tiling the slide into regions, deep learning model’s inference on these regions, and generating the heatmap for the entire slide. The advantages of BrainSec/BrainSec+ is that they can directly use the tile from the first step above, which saves data pre-processing time. As shown in Algorithm 2, we only needed to do tiling once for two models (pathology detection model and BrainSec/BrainSec+). However, FCN [42] and U-Net [29] require different data pre-processing steps, which can cause additional computation complexity for generating the distribution maps of each type pathology. On the other hand, in terms of the computation complexity of the training and inference processes, FCN [42] and U-Net [29] require a much larger GPU memory (11.9 GB) but BrainSec/BrainSec+ only use around 700 MB. Hence, we believe BrainSec and BrainSec+ are more applicable on the edge where the devices do not have a large memory. Therefore, from this perspective, we conclude BrainSec and BrainSec+ are more deployable at the edge (where the hardware resources are limited) compared to FCN [42] and U-Net [29] when we need to integrate it into a pathology related task.

### D. SEGMENTATION VISUALIZATION

**FIGURE 7.**
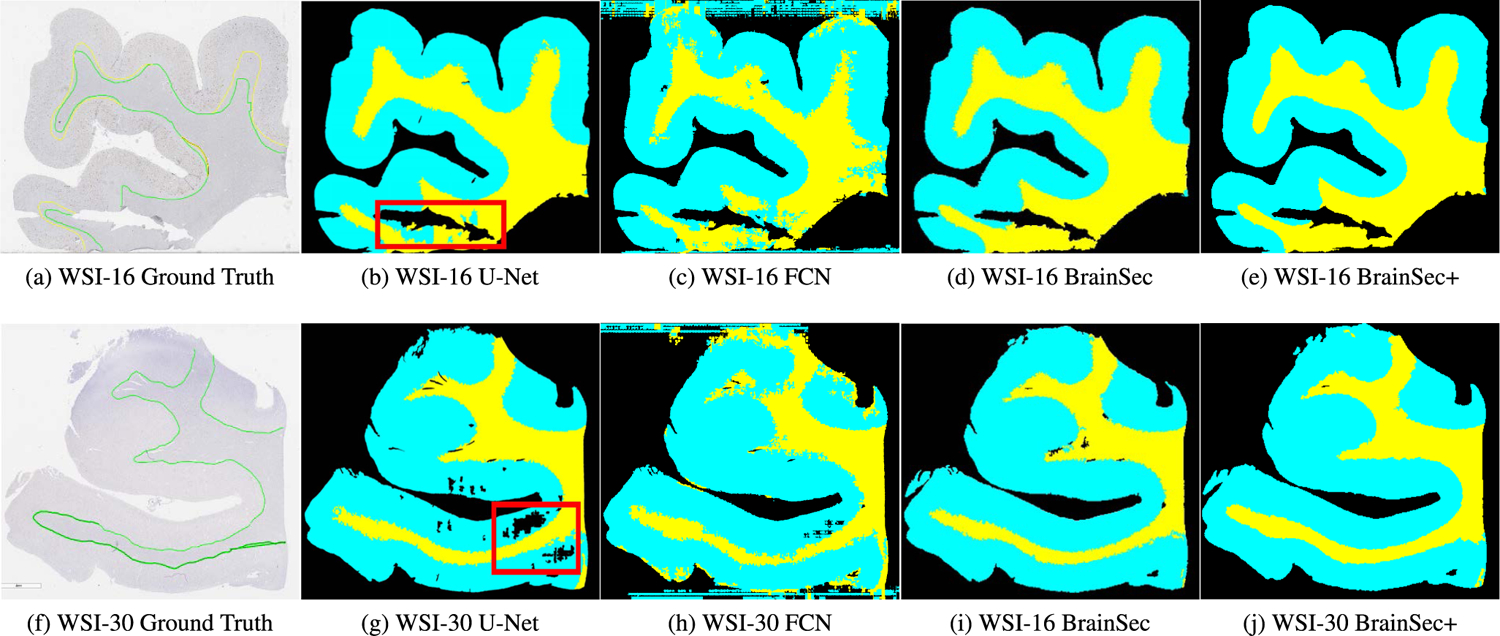
Segmentation masks visualization: WSI-16 is a AD case (top panels) and WSI-30 is a NAD case (bottom panels). GM, WM, and background are indicated by cyan, yellow, and black, respectively. The red box display areas where U-Net struggled to correctly segment tissue. In the top WSI, U-Net has trouble differentiating between WM/GM. In the bottom WSI, U-Net has trouble segmenting tissue from background.

FIGURE 7 shows the segmentation visualization of FCN, U-Net, BrainSec and BrainSec+ on hold-out AD and NAD cases separately. WSI-16 is a AD case while WSI-30 is a NAD case. In FIGURE 7, our methods segment the whole WSIs into three areas: GM, WM, and background, which are indicated by cyan, yellow, and black, respectively. The mask of FCN (FIGURE 7c, h) indicates FCN is not able to distinguish the tissue from background well (as shown in the top and bottom portions of the image where FCN detects GM when no tissue is present) while U-Net (FIGURE 7b, g), BrainSec (FIGURE 7d, i), and BrainSec+ (FIGURE 7e, j) can easily segment the tissue from background. The segmentation masks of U-Net BrainSec, and BrainSec+ are visually close to the ground-truth annotations generated by trained personnel.

### E. BRAINSEC VS. BRAINSEC+

From above sections, we show both BrainSec and BrainSec+ are able to produce consistent results. Although BrainSec requires less computation cost, its post-processing module refining the predicted masks still involves parameter tuning, which indicates we may need to tune this module manually on new datasets. To alleviate the need for such a module, we propose BrainSec+ to further automate the whole process by integrating a Neural Conditional Random Field layer into the neural networks. This additional layer could introduce computation cost but alleviate the need for a post-processing module. Hence BrainSec+ has the potential to be deployed directly on other pathology tasks without further manual tuning. On the other hand, although BrainSec+ can eliminate the need for a post-processing module, the computation cost can potentially be a limitation. In our dataset, considering BrainSec+ only achieves trivial improvements in terms of the performance but introduces heavy computation cost, we decided to use BrainSec in our Amyloid-*β* plaque quantification framework. However, if the computational resources are ample and there is a need to deploy the framework to heterogeneous settings (e.g., slides from different brain regions, scanners, or have different magnitudes) without manual post-processing, BrainSec+ will have the advantage.

### F. PATHOLOGY QUANTIFICATION WITH BRAINSEC

**FIGURE 8.**
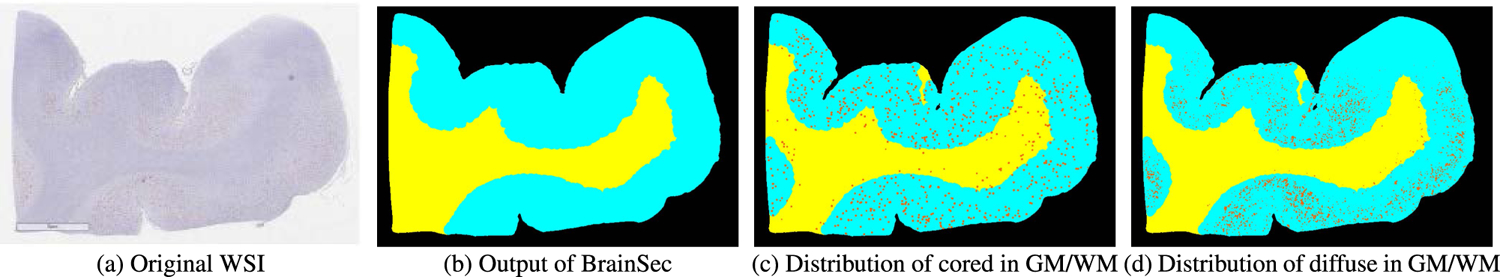
With the integration of BrainSec into the plaque identification model [12], the distribution of plaques (denoting in orange in c-d) in GM/WM can be visualized and quantified.

To investigate the compatibility of BrainSec with other pathology models, we integrated BrainSec into a recent plaque identification model [12] for quantitative pathological studies. FIGURE 8 shows one example of the output from the proposed framework: the distribution of each type of pathology can be visualized in GM and WM regions clearly. After we applied the proposed Algorithm 2, our framework detected 567 and 69 Amyloid-*β* cored in GM and WM, respectively. It also detected 1358 and 114 Amyloid-*β* diffuse in GM and WM, respectively. For this case, CAA was not detected. This is extremely advantageous for researchers to study the disease in a scalable way by automating this quantification process to relieve the manual quantification on gigapixel images. In FIGURE 8c, d, we can clearly observe that majority of pathologies appear in GM but also appear to a much lesser extent in WM, which is consistent with the findings in [11].

### G. GRAD-CAM INTERPRETABILITY

**FIGURE 9.**
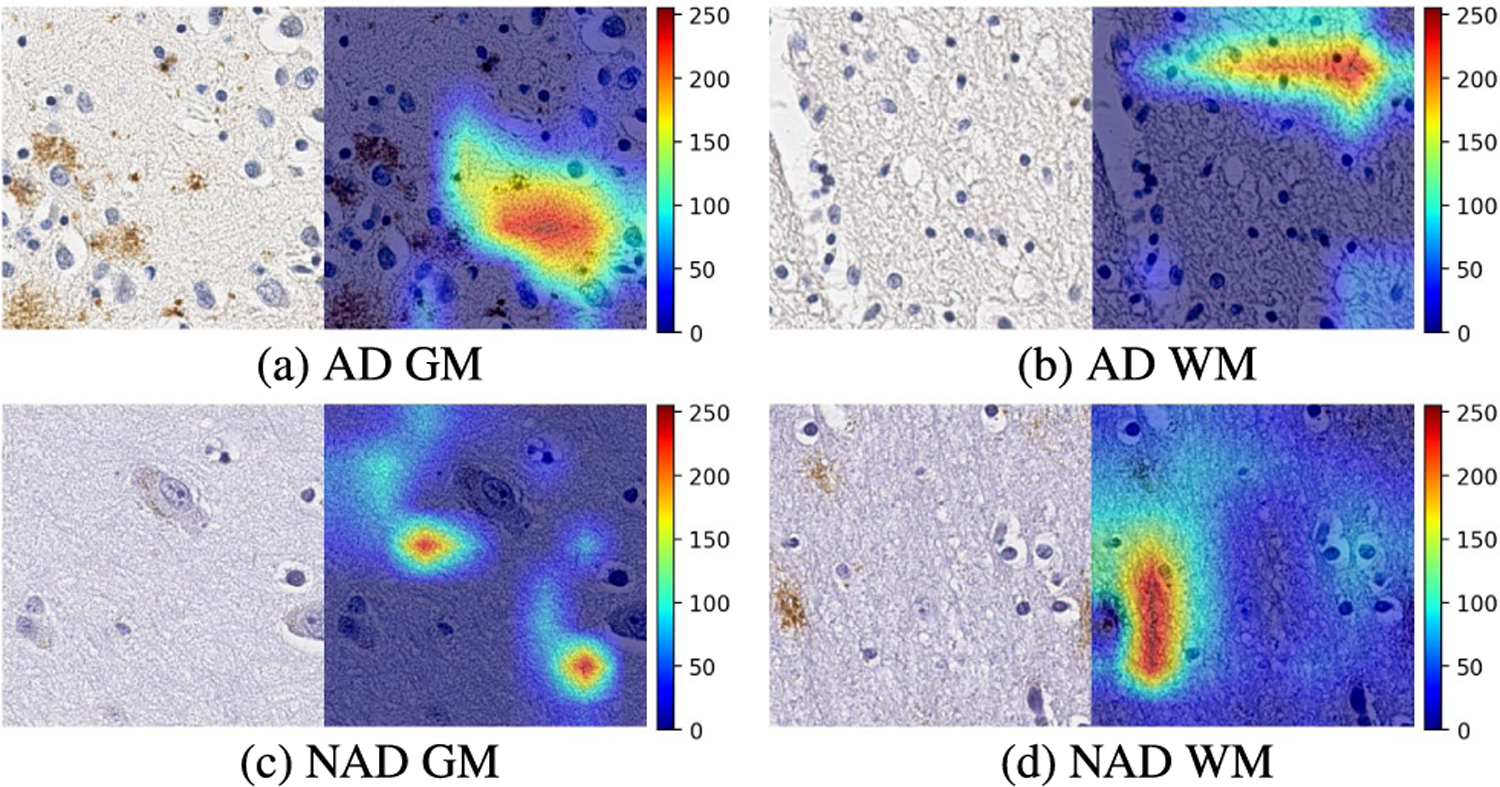
Grad-CAM on selected patches from both AD and NAD cases.

To investigate the differential morphologies corresponding to GM and WM separately, we used Gradient-weighted Class Activation Mapping (Grad-CAM) [23] to generate a coarse localization map where the relevant regions for predicting the concept are highlighted. FIGURE 9 provides visual explanations of what features BrainSec are deemed important for differentiating GM and WM for both AD and NAD cases, respectively. Grad-CAM [23] is meant for visual explanation of the deep learning models and provide a way for experts to check that the model is learning from the right sets of features to perform the segmentation or classification tasks.

From FIGURE 9, we can see the energy around Amyloid-*β* plaques (brown pixels in FIGURE 9a) and cells is relatively lower, while the highest energy is focusing on the textures of brain tissue. Based on pathology differences between GM and WM, one hypothesize is the BrainSec gravitates towards selecting areas that are composed of more randomly associated fibers (i.e. neuropil consisting of dendrites in GM, and more organized myelinated axons in WM). This is interesting as GM is also comprised of a more heterogeneous cell population (i.e. neurons and glial cells) while the predominate cell type in WM is oligodendrocytes. As a result, BrainSec not only seems to ignore pathological hallmarks of AD, such as Amyloid-*β* plaques, but also other cellular components like neuronal and glial cells. Other abnormalities such as perivascular spaces, or artifacts are also avoided. The results have the potential to prove that our CNN feature extractor is relying on the differential textures from GM and WM rather than pathologies to determine the category of the patch, additional works with greater diversity (including other areas and stains) are needed. Therefore, our feature extractor is robust to datasets from both AD cases and NAD cases, which is clinically reliable and reasonable.

### H. EVALUATION ON BROADER NEUROANATOMICAL AREAS

**FIGURE 10.**
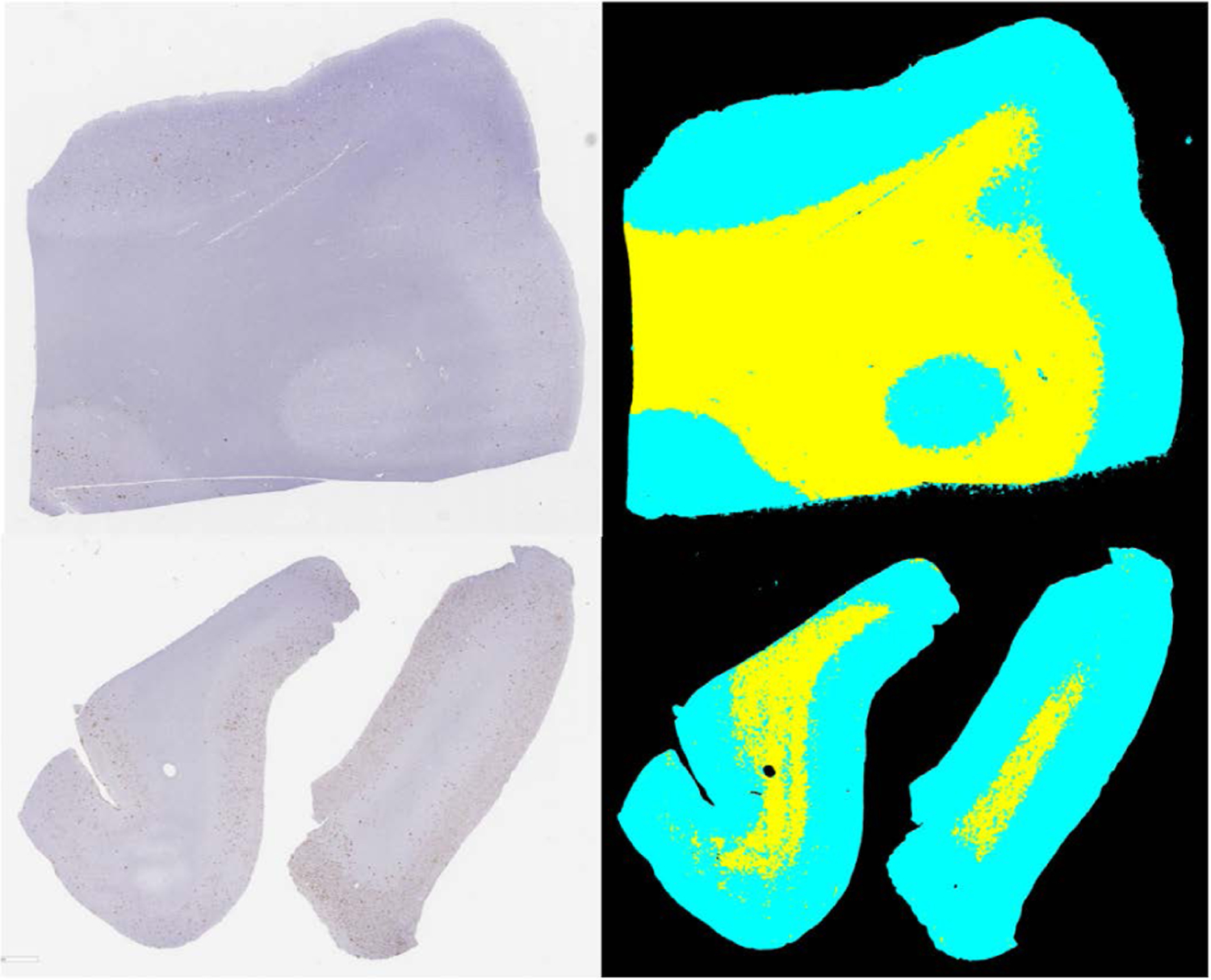
Results from running BrainSec on Frontal (top) and Parietal (bottom) WSIs.

Achieving good metrics on a medical image dataset through deep learning can sometimes be misleading. A successful model may display weak performance on similar tasks it was originally trained on if the setup or population is changed[63], which means testing BrainSec/BrainSec+ on a small dataset that contains only a single region of the brain would not guarantee us the model will perform well in all real-life scenarios. A convenience sample was prepared by selected 182 slides from three different brain regions: frontal, parietal and temporal from one institution (UC Davis), with one type of stain (4G8). This convenience sample also introduced 141 WSIs unseen by the model. Due to the large amount of data and the resource-intensive annotation process, no pixel-wise ground-truth was generated for the 182 WSIs. Meaning DICE scores and IoU metrics will not be available for this experiment. A successful prediction result would entail a well defined background/tissue boundary that visually matches the boundary observed on the WSI, as well as not presenting any errors on WM/GM segmentation when seen from 20x magnification (two examples of successful prediction results can be seen on FIGURE 10. A table comparing the original dataset with the two datasets generated by the convenience samples can be seen on (see TABLE 1).

We invited two trained personnel to visually inspect the results for our model. The results of applying BrainSec/BrainSec+ to two new neuroanatomic areas (Parietal and Frontal) showed its tendency to display stable performance when facing heterogeneous scenarios. The results displayed WM/GM segmentation that would fit the expected areas from each class upon visual inspection (as seen in FIGURE 10), even with BrainSec/BrainSec+ never seeing any tissue from the Frontal or Parietal regions in training. We also tested BrainSec/BrainSec+ on 130 Temporal WSIs. Out of these slide, 122 WSIs − **94.23%** of the dataset - displayed WM/GM segmentation that accurately match the expected areas from each region after inspection.

## VI. DISCUSSION

In this paper, we propose an automated GM/WM segmentation pipeline, named BrainSec, for gigapixel pathology images. BrainSec consists of an interpretable CNN module and a post-processing module for reducing artifacts and residues existing in WSIs as well as generate XML annotations that are helpful for neuropathologists. The final output of XML annotations can be displayed on the original-size WSIs for neuropathological studies. We show BrainSec can consistently perform well on over more than 200 slides.

We demonstrate the easy compatibility of BrainSec into other pathology models to construct quantitative pathological studies. Specifically, we integrate it into a plaque detection model [12] and construct a framework to quantify the number and density of plaques in GM/WM regions. We show BrainSec is advantageous with quantitative pathological studies in a scalable way.

Currently, our study has the limitation of needing significant computing power and time to process each WSI. Using a NVIDIA Tesla T4 GPU, it takes us about 6 hours to generate the prediction maps for WM/GM and for the Amyloid-*β* plaques for a gigapixel-level WSI. Although the existing image processing methods may not require GPU support, they cannot achieve consistent performance on the hold-out test sets (as discussed in Section II–A). Our framework is more generalizable on the hold-out cases in real-world scenarios. GPUs are required for general deep learning methods, which could be a shortcoming. On the other hand, the advantages of deep learning methods include the generalizability of the model for a scalable analysis and consistent performance, as well as reduced manual labeling efforts. After we weigh these advantages and the shortcomings, we conclude that the deep learning framework holds a lot of potential for digital pathological analysis in the future.

In the future, we plan to implement BrainSec on many different setups to continue to test its generalizability. We aim to test BrainSec on different file formats used in WSI scanning and also aim to employ BrainSec on even more neuroanatomical areas. We also must investigate how much performance metrics can be improved if our training set contains more areas than just the Temporal region.

## Figures and Tables

**TABLE 1.** Summary of the three datasets used in the study.

Dataset	Number of cases	*%* of NAD cases	Brain Areas
30-WSI	30	40.00%	Temporal
130-WSI	130	10.00%	Temporal
52-Heteroarea	26	15.38%	Frontal, Parietal

**TABLE 2.** Pixel-wise IoU comparison on the hold-out test set.

	FCN [14]	U-Net [29]	BrainSec	BrainSec*	BrainSec+
AD Back ± STD	75.46 ± 11.0	**97.32** ± **0.61**	96.79 ± 0.67	96.64 ± 1.51	96.03 ± 2.31
AD GM ± STD	71.22 ± 11.7	89.01 ± 4.92	91.10 ± 2.65	91.84 ± 2.64	**92.28** ± **2.44**
AD WM ± STD	54.69 ± 16.0	79.42 ± 7.98	82.81 ± 4.44	**85.43** ± **3.65**	84.97 ± 4.55
AD ± STD	67.12 ± 12.0	88.58 ± 4.24	90.23 ± 2.29	**91.31** ± **2.14**	91.09 ± 2.91
AD F_W ± STD	70.48 ± 9.68	90.91 ± 2.52	91.92 ± **2.32**	**92.58** ± 2.52	92.45 ± 2.74
NAD Back ± STD	78.63 ± 3.01	97.15 ± 3.30	**97.47** ± **1.39**	97.46 ± 1.98	97.08 ± 1.42
NAD GM ± STD	72.47 ± 2.91	93.75 ± 3.71	94.35 ± 1.97	**97.79** ± **1.25**	94.24 ± 1.85
NAD WM ± STD	40.70 ± 13.3	78.68 ± 8.61	79.19 ± **6.69**	81.36 ± 6.94	**83.23** ± 7.91
NAD ± STD	63.93 ± 3.54	89.86 ± 3.15	90.34 ± 2.21	91.34 ± **1.92**	**91.52** ± 2.15
NAD F_W ± STD	73.05 ± 2.65	94.31 ± 3.72	94.63 ± 2.40	**95.27** ± **1.90**	95.11 ± 2.20
Test Back ± STD	76.73 ± 8.57	**97.25** ± 1.96	97.06 ± **1.01**	97.10 ± 1.46	96.45 ± 1.95
Test GM ± STD	71.72 ± 8.93	90.91 ± 4.90	92.40 ± 2.83	**93.06** ± 2.71	**93.06** ± **2.20**
Test WM ± STD	49.09 ± 15.9	79.12 ± 7.76	81.36 ± 5.42	83.80 ± **5.28**	**84.27** ± 5.89
Test ± STD	65.85 ± 9.33	89.09 ± 3.71	90.27 ± 2.13	**91.32** ± **1.94**	91.26 ± 1.99
Test F_W ± STD	71.51 ± 7.58	92.27 ± 3.35	93.00 ± 2.62	**93.65** ± **2.58**	93.55 ± 2.68

Rows marked AD contain results on Alzheimer’s disease slides in the *hold-out* test set. Rows marked NAD contain results on non-Alzheimer’s disease slides in test set. Rows marked Test contain results on all *hold-out* test WSIs. F_W is the frequency weighted IoU score for three categories.

**TABLE 3.** DICE coefficient comparison on the hold-out test set.

Region	FCN [14]	U-Net [29]	BrainSec	BrainSec+
GM	83.21	95.17	96.03	96.18
WM	66.08	88.15	89.63	90.25

**TABLE 4.** F1-score comparison on the hold-out test set.

Region	FCN [14]	U-Net [29]	BrainSec	BrainSec+
GM	85.43	95.24	96.21	96.99
WM	69.21	88.98	90.01	91.31

## Footnotes

1
https://academic.oup.com/jnen/article/81/1/2/6495538?login=true
